# Nutritional status and associated factors of older persons in sub-Saharan Africa: a scoping review

**DOI:** 10.1186/s12877-022-03062-y

**Published:** 2022-05-11

**Authors:** Paul Obeng, Henneh Kwaku Kyereh, Jacob Owusu Sarfo, Edward Wilson Ansah, Priscilla Yeye Adumoah Attafuah

**Affiliations:** 1 Department of Health, Physical Education and Recreation, University of Cape Coast, Cape Coast, Ghana; 2 School of Nursing and Midwifery, University of Ghana, Accra, Ghana

**Keywords:** Nutritional status, Aged, Older persons, Sub-Saharan Africa

## Abstract

**Background:**

The older person population is rapidly increasing globally, including sub-Saharan Africa (SSA). Concurrently, malnutrition is also increasing among older persons in SSA, with a dearth of empirical evidence on nutritional status and associated factors among the older persons in the region to inform effective interventions to promote healthy ageing.

**Aim/Objective:**

This review assessed the nutritional status and associated factors among older persons in SSA.

**Method:**

PubMed, Google Scholar, and Cochrane Library electronic databases were searched for published articles from 2010 to 2021 using keywords and Boolean logic. Also, we carried out a free web-based search to retrieve other relevant evidence that assesses the older persons’ nutritional status. The Preferred Reporting Items for Systematic Reviews and Meta-Analyses flow chart was used to appraise the research articles that responded to the study’s research questions.

**Findings:**

Twenty studies met the inclusion criteria, from which data were extracted as findings. The malnutrition prevalence was between 6 to 54% among older persons in SSA. We found that the prevalence of malnutrition vary and could be as high as 28.4% in a low socio-economic status area. Of these studies, twelve (12) provided data on undernutrition with prevalence ranging from 2.9 to 41%;10 provided data on overweight (8.1–54.1%) and 5 on obesity (2.7–44.7%). Seventeen of the studies evaluated factors associated with malnutrition; 4 studies revealed the association between socioeconomic status and malnutrition, 7 studies reported a significant association between dietary habits and malnutrition. Four studies showed an association between educational status and malnutrition. Disease conditions associated with malnutrition were reported in four of the studies.

**Conclusion:**

This review shows that malnutrition is a problem among older persons in SSA coupled with many risk factors which should be given critical attention. We recommend nutrition education for older persons as well as the development of nutrition interventions for this vulnerable group.

## Introduction

The older population is on the rise globally, including sub-Saharan Africa (SSA) [11, 47]. Besides, the number of individuals over 60 years old is expected to double from 2015 to 2050, making the older populace more than the youth below 14 years of age [58]. Thus, morbidity and frailty are rising among older persons [3, 16, 29, 32, 47]. Moreover, the ageing process in itself affects some nutritional features. For example, the loss of sense of taste and smell, difficulty in chewing (because of loss of teeth) and poor swallowing ability, decreased secretions and weakened bowel function increase as people age [17, 20, 27, 29, 56]. Therefore, malnutrition is an important worry in older persons [17, 20, 27, 31, 56].

According to the American Society for Parenteral and Enteral Nutrition (2014), malnutrition is an imbalance between nutrient requirement and intake that negatively affects the individual’s growth. Such effects may manifest as under-nutrition, overnutrition, or micronutrient deficiencies [17, 20, 27, 31, 51].

Globally, malnutrition among older persons is rising, with undernutrition and overweight/obesity causing significant morbidity and mortality [41]. For instance, about 26% of older women and 16.3% of older men in Italy are malnourished [21]. Countries like Germany, Netherlands, and Austria have about 20.1, 18.3 and 22.5% respectively, of their healthy older population becoming either underweight or overweight/obese [4, 60].

Malnutrition manifests itself in weight loss, obesity, or clinical deficiencies in micronutrients [46]. The World Health Organisation [WHO] [63] classified nutritional status based on Body Mass Index (BMI) recordings: below 18.5 as underweight, 25.0–29.9 as pre-obesity, 30.0–34.9 as obesity class I, 35.0–39.9 as obesity class II, and above 40 as obesity class III whiles normal BMI ranges from (18.5–24.9) kg/m^2^ equivalent to a mid-upper arm circumference (MUAC) of about 23 cm [12]. Thus, good nutrition is required to enhance growth and improve the individual’s health status in the various stages of development and promote healthy ageing [31, 37]. Moreover, at a more advanced age, critical care is needed to keep the nutritional requirements of the elderly [29, 36, 49, 61]. Although individuals living in Africa are anticipated to begin living longer, ageing comes with many unbearable consequences as older people may be confronted with poor nutrition coupled with adverse health outcomes [17, 27, 29, 56].

Epidemiological studies have shown a prevalence of malnutrition between 5 and 30% in older community residents, with a significantly higher prevalence in hospitalised older people (20–60%) [59]. Also, up to half (6–48%) of older persons in SSA are underweight, and nearly a quarter (2.5–21%) are overweight [26]. There is a relationship between poverty and malnutrition [25, 52]. For instance, a cross-sectional study by Turkson and Maphepha [57] indicated that older persons were at 50% risk of malnutrition, with a greater prevalence of malnutrition in females (28%) than males (17%). Therefore, we cannot overemphasise that older persons are vulnerable to malnutrition due to sociologic, physiologic, and anatomical changes that occur along the ageing chain [4]. However, in SSA nations, the prevalence of various categories of older persons’ malnutrition and associated factors is unclear. Although Audain, Carr, Dikmen, Zotor and Ellahi [9] tried to highlight the nutritional status of older persons in SSA, they did not estimate the prevalence of malnutrition among elderly persons in SSA. Also, Audain et al. did not provide data on the various factors influencing the nutritional status of the older population in SSA. Hence, a scoping review within the sub-Saharan context is necessary to assess the general nutritional status and associated factors of older person in this area. This scoping review would influence the direction of nutritional education in older persons: reduce, change or improve the dietary pattern.

## Methodology

Our scoping review was conducted following the six-sage framework by Arksey and O’Malley [8]: identifying the research question, searching for relevant studies, selecting studies, charting the data, collating and summarizing, and reporting the results, and consulting exercises.

### Identification and selection of studies

#### Search strategy

To begin with, the authors (PO, HKK, JOS, EWA, and PYAA) carried out a vigorous literature review of published articles in three electronic databases (PubMed, Google Scholar, and Cochrane library). We further expanded, via hand, the search to include other unpolished sources. We attempted to provide answers to the questions in our review: (1) *What is the prevalence of malnutrition among the older population in SSA?; (2) What are the factors influencing malnutrition in older persons in SSA*? The search strategy was carried out using key search words and Boolean logic. Also, a free web-based search was conducted to retrieve other relevant materials which assessed the nutritional status of the elderly and its associated factors. Four keywords were used in the search strategy: (“nutritional status” OR “malnutrition” OR “nutritional assessment” OR malnourished” OR “undernutrition”) AND (“elderly” OR “aged” OR “older persons” OR “older adults” OR “geriatrics”) AND (“sub-Saharan Africa” OR “Africa”) AND (“dietary intake” OR “oral health” OR “socio-economic status”).

#### Eligibility criteria

Studies were included if conducted among the older persons 60 years and above in SSA and evaluated the prevalence and factors associated with malnutrition (underweight, overweight, and obesity). Also, the study included literature from 2012 to 2021. Studies that were not published in the English Language were excluded.

### Procedure

**Fig. 1:**
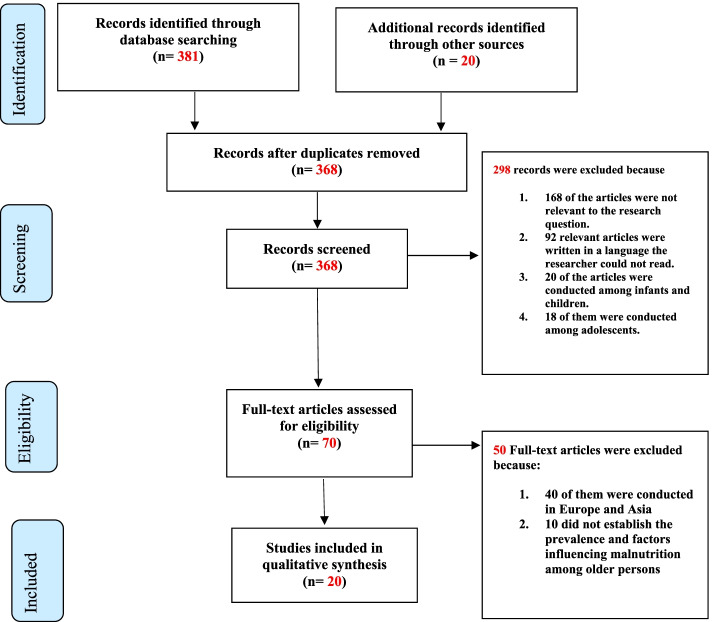
PRISMA flow chart. Source: Moher et al. [39]

At the initial stage, based on the eligibility criteria, the titles and abstracts of the identified literature were read, and only studies that were found relevant to this current study were considered. All abstracts were critically appraised to exclude articles that were not relevant to the study. Manual scanning and screening of references were done after the inclusion of a full text to include relevant literature in the review. The review was done independently by two authors (PO and HKK). The authors met and resolved differences and agreed on the studies included. A data extraction sheet was developed by the authors, including the following categories: Author, Year of publication, Study title, Country, population, study design, sample size, sampling strategy and summary of findings (Table 1). The data extraction was done independently by 3 authors (PO, HKK, JOS) and later settled differences to arrive at the final results for the study. Where there was disagreement, fourth and fifth independent reviewers (EWA, PYAA) were consulted to settle the differences. One researcher (PO), drafted the final extracted table (Results). Three authors (HKK, JOS, and EWA) read through the final draft results and made sure the findings reflected the agreed results. Additional consultations were made with subject experts to enhance the review. The Preferred Reporting Items for Systematic Reviews and Meta-Analyses (PRISMA) flow chart [39] was used to screen research articles identified and to eliminate duplicates. Figure 1 below gives details.

**Table 1:** Characteristics of studies extracted

Author	Year of publication	Study title	Country	population	Study design	Sample size	Sampling strategy	Summary of findings
Andia, Fourera, Soulemane., Mamane,& Adehossi.	2019	Evaluation of nutritional status at household in elderly assessed by Mini Nutritional Assessment (MNA)	Niger	Elderly persons	Descriptive cross sectional study	384	Multi-stage sampling method	Nutritional status was characterised by BMI and the Mini-Nutritional Assessment (MNA) tool. MNA score of ≥24 points describes a well-nourished status. A score of 17 to 23.5 points indicates a risk of malnutrition, < 17 points indicate malnutrition. The prevalence of malnutrition was 7.8% which increased with the elderly from 5.8% (60 to 74 years) to 6.7% for more than 85 years old.
Jesus, Guerchet, Pilleron, Fayemendy, Mouanga, Mbelesso,& Desport,	2017	Undernutrition and obesity among elderly people living in two developing cities: prevalence and associated factors in the EDAC study	Central African Republic (CAR), Congo	Aged person 65 years and above	Cross-sectional study	990	Random sampling	BMI < 18.5 was considered undernutrition, between 18.5 and 24.9 is considered normal weight, a BMI between 25.0 and 29.9 overweight, and > 30.0 obesity. The study was conducted in two countries. While undernutrition in Congo was lower than in CAR (9.5% vs 29.5%), obesity was rather high in Congo (14.6%) than in CAR (2.7%). Socio-economic actors and food consumption were associated with malnutrition.
Dorcah Kwamboka Okinyi	2021	Nutritional Status Among the Geriatrics in Nyamira County, Kenyaperson	Kenya	Persons 60 years and above person	Cross-sectional study	230	multi-stage sampling technique	BMI and MNA were used to determine the nutritional status of participants. Based on the BMI measure, person31% were malnourished, and 42% were at risk. MNA scores exhibited that (54%) were malnourished, 29% were at risk and 17% were well nourished. Marital status (χ2 = 27.7699, *P* < 0.01), level of education (χ2 = 37.260, *P* < 0.001) and level of stress (χ2 = 57.712, *P* < 0.001) were predictors of malnutrition.
Alphas, C., Atuhairwe, C., Amongin, D., & Taremwa, M. I.	2018	Determinants of nutritional status among geriatric populations in Kween District, Eastern Uganda	Uganda	Older persons	Cross-sectional survey	250	Consecutive sampling approach	BMI and Mid-Upper Arm Circumference (MUAC) were used to determine the nutritional status of participants. The prevalence of malnutrition was evaluated to be 6%. The determinants of geriatric malnutrition include: age, weight, level of education, number of children, declined food intake, and as well neuropsychological problems.
Adebusoye, L.A., Ajayi, I. O., Dailo, M. D., & Ogunniyi, A. O.	2012	Nutritional status of older persons presenting in a primary care clinic in Nigeria	Nigeria	Older persons 60 years and above	Cross-sectional descriptive study	500	Simple random sampling	A validated and standardised Mini-Nutritional Assessment (MNA) tool was used to assess the nutritional status of participants. BMI, mid-arm and calf circumference, and weight were used to determine the nutritional status of participants. The prevalence of undernutrition was 7.8%, while that of overweight was 54.1%. The aged with the oral problem were more likely to suffer undernutrition.
Robb, L., Walsh, C. M., Nel, M., Nel, A., Odendaal, H., & van Aardt, R.	2017	Malnutrition in the elderly residing in long-term care facilities: a cross-sectional survey using the Mini Nutritional Assessment (MNA) screening tool	South Africa	Older persons residing in a long-term care facility	Cross-sectional	162	Simple random sampling	The nutritional status of participants was assessed using the MNA questionnaire. BMI, mid-upper arm circumference (MUAC) and calf circumference (CC) were used to measure malnutrition. Participants from the low socio-economic area had a higher prevalence of malnutrition (3.2%) than those in high socio-economic areas (11.3%), while the dietary intake was significantly associated with malnutrition.
De Rouvray, C., Jesus, P., Guerchet, M., Fayemendy, P., Mouanga, A. M., Mbelesso, P.,…& Desport, J. C.	2014	The nutritional status of older people with and without dementia living in an urban setting in Central Africa	The central African Republic and the Republic of Congo	Aged over 65 years	Cross-sectional survey	1016	Cluster sampling	BMI was used to determine the nutritional status of participants. The prevalence of undernutrition was 19.2%. Compared with healthy people, persons with dementia had an increased prevalence of undernutrition (32.0% vs 17.7%). Poor diet was as well associated with being underweight.
Aganiba, B. A., Owusu, W. B., Steiner-Asiedu, M., & Dittoh, S.	2015	Association between lifestyle and health variables with nutritional status of the elderly in the northern region of Ghana	Ghana	Aged 65 years and above in the northern region	Cross-sectional study	400	A multi-stage sampling technique	Using BMI (in kg/ m^2)^ as an indicator for nutritional status, 18.0% of the participants were underweight, 60.5% had normal weight, and 21.5% were overweight. Alcohol consumption and sight problems were found to have a significant negative association with BMI. More males (19.8%) than females (16.6%) had a BMI less than 18.50 kg/m^2^. On the other hand, more females (about 25%) than males (17%) were overweight.
Tessfamichael, D., Gete, A. A., & Wassie, M. M. De Rouvray	2014	High prevalence of undernutrition among elderly people in northwest Ethiopia: a cross sectional study	Ethiopia	All old people 65 years and above in Gonda town	Cross-sectional study	757	Two-stage cluster sampling method was used	BMI was used to measure the nutritional status of participants. The study indicated a high prevalence of undernutrition (21.9%) among the elderly. Old age range, gender, educational status, dietary diversity and socio-economic status were found to be factors affecting undernutrition.
Turkson, R. K. D., & Maphepha T. F.	2019	Using novel nutritional assessment tool to assess the nutritional status of the elderly in Mazenod: the case of old-age pension scheme in Lesotho	Lesotho	Elderly 70 years and above	Cross-sectional qualitative and quantitative study	60	Random sampling	The Novel Nutritional Status Assessment tool was used to assess the nutritional status of elderly persons. 50% of the elderly are at risk of malnutrition. The prevalence of malnutrition in males (17%) is lower than in females (28%).
Maila, G., Audain, K., & Marinda, P. A.	2021	Association between dietary diversity, health and nutritional status of older persons in rural Zambia	Zambia	Older persons in Milenge	Cross-sectional, descriptive study	135	Random sampling	Anthropometric measurements (weight and height) were used to determine the prevalence of malnutrition among the respondents by calculating the body mass index (BMI). Thirty-four percent of the participants were underweight, and 8.1% were overweight. More men had severe underweight whereas the opposite was observed in women. Thus, more women were either overweight or obese. Dietary intake was significantly associated with malnutrition.
Abdulgafar Lekan Olawumi, Bukar Alhaji Grema, Abdullahi Kabir Suleiman, Yakubu Sule Omeiza, Godpower Chinedu Michael, Abdulrahman Shuaibu	2021	Nutritional status and morbidity patterns of the elderly in a Northwestern Nigerian hospital: A cross-sectional study	Nigeria	Person Patients 60 years and above	Descriptive cross-sectional study	348	Systematic random sampling	BMI, mid-arm circumference and calf circumference (CC) were used to predict the nutritional status of participants. Prevalence of malnutrition was 25.3%, and those at risk of malnutrition were 56.6%. Advanced age, lack of formal education, low monthly income, chronic respiratory diseases, physical inactivity and duration of chronic disease for more than 10 years were the predictors of malnutrition.
Cheserek, M. J., Tuitoek, P. J., Waudo, J. N., Msuya, J. M., & Kikafunda, J. K.	2012a	Anthropometric characteristics and nutritional status of older persons in the Lake Victoria Basin of East Africa: region, sex, and age differences: original research	Kenya, Uganda and Tanzania	Older persons aged 60 years and above	Cross-sectional study	227 men and 310 women	–	Measures used were weight, height, arm span, mid-upper-arm circumference (MUAC) and triceps skinfold thickness (TSF). Overall underweight (BMI < 18.5 kg/m2) was 26.4, 58.3% were normal (BMI 18.5–24.9 kg/m2), 10.8% were overweight (BMI 25–29.9 kg/m2), and 4.5% were obese (BMI ≥ 30 kg/m2). Older men (29.5%) were significantly more underweight (*p*-value< 0.05) than older women (24.2%), overweight (12.5%) and obesity (6.8%) were significantly higher in older women
Herve Hien, Abdramane Berthe, Maxime Koine Drabo, Nicolas Meda, Blahima Konate, Fatoumata Tou, Fatoumata Badini-Kinda and Jean Macq	2014	Prevalence and patterns of multimorbidity among the elderly in Burkina Faso: cross-sectional study	Burkina Faso	Elderly persons 60 years and above	Cross-sectional study	389	Random clusters	BMI was used to determine the nutritional status of older persons. About 39% of the older persons were malnourished. Those aged ≥70 had significantly more malnutrition (50% vs. 31%, *P* = 0.0003) than those aged 60–69.
B. Blankson, A. Hall	2012	The Anthropometric Status Of Elderly Women in Rural Ghana and Factors Associated With Low BMI.	Ghana	Elderly women aged 60 to 92 years	Cross-sectional survey	59	-	The weight, height, half arm span and mid-upper arm circumference (MUaC) of each woman were measured; body mass index (BMI) and body mass for arm span (BMa) were calculated. 41% of women were underweight, and 16.9% were overweight or obese. Factors associated with a low BMI were: age (*P* = 0.001), chewing tobacco (*P* = 0.002), drinking alcohol (*P* = 0.012), a visual acuity score of < 30% (*P* = 0.038), using a walking aid (*P* = 0.016) and the number of children who gave the women cash (*P* = 0.005).
Cheserek, M. J., Waudo, J. N., Tuitoek, P. J., Msuya, J. M., & Kikafunda, J. K	2012b	Nutritional Vulnerability of Older Persons Living in Urban Areas of Lake Victoria Basin in East Africa: A Cross Sectional Survey	Kenya, Uganda, and Tanzania	Persons aged 60 years and above	Cross-sectional study	128	Systematic random selection	BMI was used as a nutritional status indicator by comparison to WHO cut-offs. The prevalence of underweight was 16.5%, with men (24.1%) being significantly more likely to be underweight (*P* < 0.05) than women (12.3%). Overall, 61.2% had normal body mass indices, 13.2% were overweight, and 9.1% were obese. Inadequate food access, poor health, living arrangements, and poor eating patterns were the main nutritional risk factors
Faith Agbozo, Joyce Amardi-Mfoafo, Helen Dwase, Basma Ellahi	2018	Nutrition knowledge, dietary patterns and anthropometric indices of older persons in four peri-urban communities in Ga West municipality, Ghana	Ghana	Elderly aged 60–70 years	cross-sectional survey	120	Purposive Sampling technique	BMI was according to WHO age and sex-specific guidelines to determine the nutritional status of the aged. Underweight was 10%, while 21.7% were overweight or obese (16.6%). Positive insignificant corrections existed between knowledge and nutritional status (*r* = 0.261) and diet quality (*r* = 0.415). However, a strong significant (*p* = 0.027) positive correlation (*r* = 0.699) existed between diet quality and nutritional status
Afolabi, W. A. O., Olayiwola, I. O., Sanni, S. A., & Oyawoye, O.	2015	Nutrient Intake and Nutritional Status of the Aged in Low Income Areas of Southwest, Nigeria	Nigeria	Persons aged 58–99 years	cross-sectional and descriptive	140	A multistage sampling technique	BMI was used to determine the nutritional status of the aged. The prevalence of underweight was low (2.9%). Being overweight was high (20% for men and 22.8% for women). Weight and BMI are influenced by the energy intake of the men (*r* = 0.439, *p* = 0.008); (*r* = 0.352,*p* = 0.038). There was a high prevalence of overweight which coexists with underweight among the aged
GT Fadupin	2012	Social Support, Environmental Condition and Nutritional Status of the Elderly in Ibadan	Nigeria	Persons 60 years and above		150		BMI was used to classify the nutritional status of the aged. The BMI of the respondents indicates that 48.0, 7.3, and 44.7% of the respondents had normal weight, underweight, and overweight or obesity, respectively
Ogechi Chinyere Nzeagwu	2016	Evaluation of Nutritional Status Using Anthropometry and Biochemical Indices of Community Dwelling Older Persons in Nigeria	Nigeria	older persons of 65 years and above	cross-sectional	600	Multi-stage random sampling	BMI and MUAC were used to determine the nutritional status of participants. Most (62.7%) had normal BMI, while 21.33% were overweight. About 75.6% had normal MUAC, and 24.33% were malnourished.

## Results

### Search results

We used the PRISMA flow chart was used to assess the research articles included in the study (Fig. 1). We conducted an updated literature search in March 2022 where 401 literature were retrieved. We further examined the abstracts of the retrieved literature for duplication and eligibility and later excluded 381. Out of the retrieved literature: 153 were from Google Scholar, 120 from Pubmed, 108 from Cochrane library and 20 from hand search. After we had screened the title and abstract, 70 articles were eligible for full-text evaluation. Subsequently, twenty (20) studies qualified for final analysis. Figure 1 presents a flow diagram detailing the list of all the searches. Table 1 summarises the characteristics of the included studies.

### Findings

The included articles were studies conducted among older persons 60 years and above, conducted in SSA, assessed the nutritional status and associated factors of the older persons. The reviewed literature were studies published from 2012 to 2021. Five of the studies were conducted in Nigeria, two from the Central African Republic (CAR) and Congo, three from Ghana, three from Kenya and Uganda, with only one study each conducted in Burkina Faso, Zambia, and Lesotho, Ethiopia and South Africa each. All the studies, 20 (100%), employed a descriptive cross-sectional design. Five (41.7%) of the studies employed simple random sampling techniques to sample participants (Table 1). Seventeen (17) studies assessed the nutritional status of older persons, while 3 studies examined the factors associated with the nutritional status of older persons. Based on our research questions, two themes were derived from the reviewed studies: (1) Prevalence of malnutrition among older persons in SSA and (2) factors influencing malnutrition among older persons in SSA.

### Prevalence of malnutrition among the older persons

Our review summarises evidence from the included studies on the prevalence of malnutrition among older persons. Nineteen) studies assessed the prevalence of malnutrition among older persons. One of these studies found a 7.8% of malnutrition prevalence among older persons [6]. Also, one study recorded the prevalence of malnutrition using BMI and Mini-Nutritional Assessment (MNA) tool measures to be 31 and 54%, respectively among older persons [44]. Other studies also recorded the prevalence of malnutrition in older persons to be 6% [5] and 25.3% [1, 45] and 39% [28]. Of these studies, 12 provided data on undernutrition, with prevalence ranging from 2.9 to 41% ([1–4, 12, 14, 15, 19, 24, 30, 38, 55]). Ten studies provided data on overweight, with the studies recording 54.1% [1], 21% [3] and 8.1% [38] prevalence of overweight. Obesity was assessed in 5 studies, ranging from 2.7 to 44.7% [12, 14, 15, 24, 30] (see Table 1).

### Factors influencing malnutrition in older persons

This study summarises evidence on the factors influencing malnutrition in older persons in SSA (Table 1). Seventeen studies reported on the various factors associated with malnutrition; four studies revealed the association between socio-economic status and malnutrition [30, 45, 48, 55], whereas seven studies were on dietary habits ([5, 15, 19, 30, 38, 48, 55]). Also, four studies showed a significant association between educational status and malnutrition [5, 44, 45, 55]. Disease conditions related to malnutrition were assessed in four studies [1, 5, 19, 45] (see Table 1).

## Discussion

This study aimed at determining the nutritional status and associated factors of older persons with SSA. We explored the prevalence of malnutrition and factors influencing the nutritional status of older persons in SSA.

### Prevalence of malnutrition

Our review summarises evidence from the included studies on the prevalence of malnutrition among older persons. Nineteen (19) studies assessed the prevalence of malnutrition among older persons. These studies recorded prevalence ranging from 6 to 54%. Of these studies, twelve (12) studies provided data on undernutrition, with prevalence ranging from 2.9%3.2 to 41%. Ten studies provided data on overweight, with a prevalence of 8.1 to 54.1%. Obesity was assessed in five studies, ranging from 2.7 to 44.7%.

The findings of this current review show a high prevalence of malnutrition ranging from 6 to 54% among the elderly population in SSA. This high prevalence of malnutrition among older persons may expose them to cardiovascular disease (CVDs), including stroke and ischemic heart disease, potentially affecting their quality of life (QoL) [40]. In agreement with our findings, a transversal descriptive study by Andia et al. [6] shows a 7.8% malnutrition prevalence among the elderly. A similar finding of 6% prevalence was recorded in a cross-sectional study by Alphas et al. [5] from Kween district, a suburb of Eastern Uganda. However, Alphas et al. realised that more females (28%) were malnourished compared with men (17%). Moreover, similar to our findings, another cross-sectional survey in South Africa found a prevalence of 11.3% of malnutrition among older persons [48]. This higher prevalence of malnutrition may increase mortality and morbidity among the older person population in SSA. Contrary to our findings, Robb et al. [48] reported a lower prevalence of malnutrition among older persons in high socio-economic status areas (3.2%). According to Davison et al. [18], older persons in high socio-economic areas can afford gym services and healthy foods such as fruits and vegetables, perhaps the reason for the disparities in the nutritional status between the poor and the rich older persons.

In line with our findings, Andre et al. [7] determined the BMI of older persons where they recorded a malnutrition prevalence of 31% among older persons. However, Andre et al. further recorded the prevalence of malnutrition to be 54% using the NMA scale for the same population. This shows that the prevalence of nutritional status may vary based on the nutritional assessment tool used. In line with our findings, dwelling in rural Louzi, Congo, approximately 57.8% of the elderly were at risk of malnutrition, while 28.4% were malnourished [7]. Accounts from these studies show that the prevalence of malnutrition was low in high socio-economic status regions.

Furthermore, our review found that the prevalence of undernutrition among older persons ranged from 3.2 to 30.4% in SSA. Underweight among older persons puts them at risk of experiencing osteoporosis [10]. This condition may expose them to experience a fracture in a case of a little fall which may affect their health and QoL [35]. According to Jesus et al. [30], a nutritional assessment conducted in the Central Africa Republic (CAR) among older persons found that 29.5% were undernourished. This finding was consistent with Boscatto, Duarte, Coqueiro, and Barbosa [13], who recorded the prevalence of undernutrition among older women to be 27.3%. However, this higher prevalence of undernutrition was attributed to the lower socio-economic status of the older population in both studies. Though these prevalences were high ([30] [29.5%]; [13] [27.3%]), other studies were found to be consistent with these findings [3, 11, 19]. In most of the communities that recorded a high prevalence of undernutrition, the socio-economic status was low to an extent. Contrary to this, other studies found quite low prevalence. For example, in a study conducted by Adebusoye et al. [1] in Nigeria, the prevalence was recorded to be 7.8%. Poor oral health, food insecurity and poor dietary intake were associated with being underweight in this population.

Also, we found the prevalence of overweight in older persons in SSA ranging from 8.1 to 54.1%, with an obesity prevalence of 2.7 to 44.7%. Being overweight or obese can have a serious impact on health. Overweight and obesity in older persons may lead to serious health consequences such as CVDs (mainly heart disease and stroke), type 2 diabetes, musculoskeletal disorders like osteoarthritis, and some cancers [endometrial, breast and colon] [34, 62]. These conditions may cause death, substantial disability and affect the QoL among older persons in SSA. Compared with undernutrition, overweight and obesity have higher SSA prevalence, especially among older persons [53]. In Nigeria, a cross-sectional study by Adebusoye et al. [1] revealed that 54.1% of the study population had BMI greater than 25 kg/m^2^. However, in a recent study by Okafor and Adepoju [43], the prevalence of overweight and obesity were 22.8 and 10.0%, respectively, among the elderly population. Though these prevalences were high, they cannot match the earlier findings by Adebusoye et al. [1]. It is not clear what accounted for the wider gap in the prevalence of these two studies, even though they were all conducted in Nigeria. Thus, there is a need for further investigation to explore this situation.

A comparison study in two countries, the Republic of Congo and the CAR, showed that while undernutrition was higher among the older persons in CAR than in Congo, the reverse was overweight and obesity [30]. It has not been clear what might have influenced the difference in overweight between CAR and Congo, the economic status together with the standard of living in these two countries cannot be overlooked. Thus, more studies need to be conducted to probe further the factors influencing the nutritional status of older persons in CAR and Congo.

### Factors associated with malnutrition

Seventeen (17) studies also reported on the various factors associated with malnutrition; four studies revealed the association between socio-economic status and malnutrition, whereas seven studies were on dietary habits. Also, four studies showed a significant association between educational status and malnutrition. Disease conditions related to malnutrition were assessed in four of the studies. The review revealed a clear association between socio-economic status and malnutrition of the older persons in SSA. Most African adults experience poverty, poor health care access and services, and poor diet in old age [53]. For instance, it is indicative that older individuals in urban centres or with high socio-economic status were more likely to be overweight and obese. On the contrary, those in the rural areas or with low socio-economic status were undernourished in most cases [11, 30, 48].

Also, we found a significant association between the educational status of older persons and malnutrition. Affirmatively, according to Alphas et al. [5], the level of formal education an individual attains is a key determinant of employment, utilisation of health, and educational services. This defines the socio-economic status of the individual, which influences the nutritional status of the aged. In line with our findings, other studies also found a significant association between educational status and malnutrition [44, 45, 55].

Furthermore, our review found that consumption of an unbalanced diet (poor diet), decline to eat by the older persons and poor dietary patterns influenced malnutrition among the older persons in SSA. The primary outcome of this habit is underweight, overweight or obesity. Steyn and Mchiza [53] support this earlier finding and that nutrient intake together with age, gender, and increased energy fat was significantly associated with overweight and obesity.

Also, In comparison, women showed a higher prevalence of overweight than men, and in urban than rural areas, which also progress with advancing age [23]. Changes in dietary behaviour and sedentary lifestyle coupled with less physical activities make the elderly at higher risk of being overweight or obese than younger adults. However, older women have been found to engage in a more sedentary lifestyle as compared to older men [22, 54]. This may have influenced the higher prevalence of overweight and obesity among older women than older men. Obesity and overweight among older persons may expose them to risks of CVDs such as hypertension and diabetes [50].

In addition, we found that older persons with oral problems were more likely to suffer from undernutrition. According to Adebusoye [1], oral problems significantly affect the nutritional status of older persons. Poor oral health affects an individual’s ability to masticate meals and crush hard meat. This may result in loss of appetite or the need to take meals. To this effect, oral health has been negatively associated with malnutrition, resulting in older persons being underweight [33].

## Limitations

This study has provided insight into the nutritional status and associated factors of older persons in SSA. However, there are a few limitations of the study. Firstly, our literature search was restricted to studies published in English for practical reasons. We may have missed some relevant literature. Also, due to restrictions of some databases, we focused our search on only three databases and freehand search; we may have missed other relevant literature in our review.

## Conclusion

Malnutrition among older persons in SSA is high, with a higher prevalence of undernutrition, overweight and obesity. A higher prevalence of undernutrition implies that older persons in SSA are at risk of developing osteoporosis and a weak immune system accompanying complications. Also, the higher prevalence of overweight and obesity among the older persons in SSA implies that the older person population in the continent are at risk of developing CVDs and diabetes. The consequences of both undernutrition, overweight, and obesity may affect the QoL of older persons if critical attention is not given to their nutritional needs. Older persons with low socio-economic status, poor dietary habits, and those experiencing disease conditions are more likely to be malnourished. Suppose adequate measures are not implemented to improve older persons’ socio-economic status and dietary habits. In that case, they are likely to experience morbidity and mortality, which may burden the countries’ health care systems and the economy in SSA. Also, SSA’s efforts to attain sustainable development goal three, to ensure healthy lives and promote well-being for all ages, may be hindered.

## Recommendations

This scoping review established that older people even though with increased risk of malnutrition, and most of them being malnourished [4, 26, 42, 57, 59], there is not much attention given to their nutritional and related needs. It will be appropriate if intervention driven quantitative studies are conducted to assess the nutritional requirement of older persons. More importantly, there should be a nutritional policy that will improve older persons’ dietary habits. Caregivers of older persons need nutrition education to increase awareness of proper nutritional care. Also, Financial support systems should be established and made available to support older persons.

## Directions for future studies

Based on our findings, further exploratory studies among individual SSA countries would likely yield key evidence on the current case of malnutrition among older persons. With a suggestive growing prevalence of malnutrition among older persons with low socio-economic statuses, the role of social support and social interventions for older persons have to be explored by researchers in SSA.

## Directions for policies

Governments and stakeholders in SSA should implement effective social and health promotive interventions to address the nutritional health needs of older persons. These interventions should pay attention to their socio-cultural perspectives and socio-economic statuses. As the world focuses on attaining the Sustainable Development Goals, governments and stakeholders in SSA should enact policies to address older person care and nutritional health needs.

## Conflicting interest

The authors declare that they have no known conflicting personal or financial interests regarding the conduct of this study that could have affected the findings reported.

## Data Availability

All data generated or analysed during this study are included in this published article.
